# Whole-genome sequencing of *Burkholderia pseudomallei* from an urban melioidosis hot spot reveals a fine-scale population structure and localised spatial clustering in the environment

**DOI:** 10.1038/s41598-020-62300-8

**Published:** 2020-03-25

**Authors:** Audrey Rachlin, Mark Mayo, Jessica R. Webb, Mariana Kleinecke, Vanessa Rigas, Glenda Harrington, Bart J. Currie, Mirjam Kaestli

**Affiliations:** 1 Menzies School of Health Research, Charles Darwin University, Darwin, Northern Territory 0811 Australia; 2 Royal Darwin Hospital and Northern Territory Medical Program, Darwin, Northern Territory 0811 Australia; 3 Research Institute for the Environment and Livelihoods, Charles Darwin University, Darwin, Northern Territory 0811 Australia

**Keywords:** Microbial genetics, Bacterial genetics, Infectious-disease epidemiology

## Abstract

Melioidosis is a severe disease caused by the environmental bacterium *Burkholderia pseudomallei* that affects both humans and animals throughout northern Australia, Southeast Asia and increasingly globally. While there is a considerable degree of genetic diversity amongst isolates, *B. pseudomallei* has a robust global biogeographic structure and genetic populations are spatially clustered in the environment. We examined the distribution and local spread of *B. pseudomallei* in Darwin, Northern Territory, Australia, which has the highest recorded urban incidence of melioidosis globally. We sampled soil and land runoff throughout the city centre and performed whole-genome sequencing (WGS) on *B. pseudomallei* isolates. By combining phylogenetic analyses, Bayesian clustering and spatial hot spot analysis our results demonstrate that some sequence types (STs) are widespread in the urban Darwin environment, while others are highly spatially clustered over a small geographic scale. This clustering matches the spatial distribution of clinical cases for one ST. Results also demonstrate a greater overall isolate diversity recovered from drains compared to park soils, further supporting the role drains may play in dispersal of *B. pseudomallei* STs in the environment. Collectively, knowledge gained from this study will allow for better understanding of *B. pseudomallei* phylogeography and melioidosis source attribution, particularly on a local level.

## Introduction

*Burkholderia pseudomallei* is an environmental Gram-negative bacillus and the causative agent of melioidosis, a potentially fatal infection of humans and animals^1^. Regions of high *B. pseudomallei* endemicity predominantly include Southeast Asia and northern Australia, though the bacterium is also increasingly found in other tropical regions including the Indian subcontinent, Africa and the Americas^2^. Melioidosis is typically considered noncommunicable with direct person-to-person transmission and zoonotic disease being remarkably rare^3,4^. Nearly all *B. pseudomallei i*nfections are caused by a single direct exposure event to contaminated soil or surface water in the environment and individual cases of melioidosis are typically the result of infection by different strains of the bacterium^5^.

A limited geographic dispersal of *B. pseudomallei* strains has also been identified and the bacterium is now recognised as being ecologically established and spatially clustered in the environment^6–8^. This is in spite of the frequent opportunities *B. pseudomallei* has to spread within the water table, via agricultural and migratory animals, or in transported soil^6,9–11^. This restricted geographical distribution has resulted in distinct genetic populations of the bacterium, which are evident despite high levels of gene recombination and sequence type (ST) diversity^9,12,13^. While the high rate of genetic diversity and recombination previously hindered the examination of *B. pseudomallei* populations using traditional typing methods the development of whole-genome sequencing (WGS) has facilitated the examination of genetic populations of the bacterium on a fine-scale^8,12,14,15^. This has allowed for population and evolutionary inferences to be made on both a global and local level. WGS, in conjunction with multi-locus sequence typing (MLST) data, has revealed distinct geographical partitioning between Australian and Southeast Asian isolates separated by Wallace’s Line^12,13,16^. Additionally, no shared environmental STs have been identified within the Northern Territory and adjacent Queensland, northern Australia, with distinct *B. pseudomallei* population structures identified in the two regions using Bayesian MLST-based analysis^9^.

While more than 450 MLST types have now been classified in the Northern Territory, Australia, (https://pubmlst.org/bpseudomallei/) five ST’s have been shown to comprise 90% of the overall sequence type abundance in the Darwin region of the Northern Territory, Australia and the maximum geographic distance identified between environmental isolates of the same strain is typically in the range of 50 linear kilometres^6,7,17^. This again supports the idea that despite the high degree of diversity, populations of *B. pseudomallei* are ecologically established and not widely dispersed in the environment. Despite the high degree of ST diversity in the Northern Territory environment a distinct genetic population structure of the bacterium has yet to be identified there.

Within the “Top End” of the Northern Territory, Australia, *B. pseudomallei* is frequently isolated in the environment and there have been more than 1,150 culture-confirmed human cases of melioidosis diagnosed since 1989^5,6,18^. The coastal capital city of Darwin (12°S latitude) has by far the highest incidence of melioidosis reported for any urban environment globally, with rates up to 50 per 100,000 annually^19^. Apart from soil, *B. pseudomallei* is also frequently detected in water. A third of tested unchlorinated rural water bores, of which there are over 5,000 in the Darwin surrounds, have been shown to be *B. pseudomallei* positive^20^ and these have been implicated in clusters of melioidosis infections in the past^4,21,22^. Moreover, studies into seasonal disease correlates in northern Australia have demonstrated an association between the frequency of cases and the nature and timing of rainfall-related events^23^. Contaminated water and *B. pseudomallei*’s movement within the water table have recently been demonstrated as a potentially significant environmental reservoir and distribution tool for the bacterium there^11,24^. Environmental isolates recovered from groundwater seeps in Townsville, Queensland, Australia were later linked to clinical isolates using molecular typing^11,24^.

Since storm-water is known to capture and leach what is in the land, including particles, contaminants and bacteria, it may provide a more appropriate indication of catchment and *B. pseudomallei* distribution, as the bacterium is able to further disperse along drainage lines^25–27^. As a result of *B. pseudomallei’s* heterogeneous distribution in the environment, the identification of new areas endemic for melioidosis may be more effectively determined by analysing surface runoff and storm-water than by the analysis of random soil samples^11,27,28^.

The aim of this study was to examine the distribution and local population structure of *B. pseudomallei* in urban Darwin, Northern Territory, Australia targeting public parks and drains. We sampled soil and land runoff throughout the city and did WGS analysis on cultured isolates. By combining genome variant analysis and Bayesian clustering methods we examined the local phylogenetic structure of *B. pseudomallei* and used spatial hot spot analyses to examine the spatial clustering of the bacterium on a small geographic scale. We hypothesised that *B. pseudomallei* would be well-established throughout the urban Darwin environment and there would be a considerable amount of ST diversity amongst isolates, particularly at drain catchment sites. However, we predicted that genetic spatial clustering of *B. pseudomallei* would not occur on such a localised geographic scale.

## Results

### Presence of *B. pseudomallei* in urban Darwin drains

From the 42 drain sites surveyed in the first sampling round in the early wet season, 2016, 19 (19/42, 45.2%) had water available for collection. Five of these 19 sites only contained enough water to collect one replicate. Of the 47 total water samples collected, five samples (5/47, 10.6%) were qPCR and/or culture positive at three sites (3/19, 15.8%). Two of the samples (2/47, 4.3%) were qPCR positive only at two sites (2/19, 10.5%).

**Figure 1:**
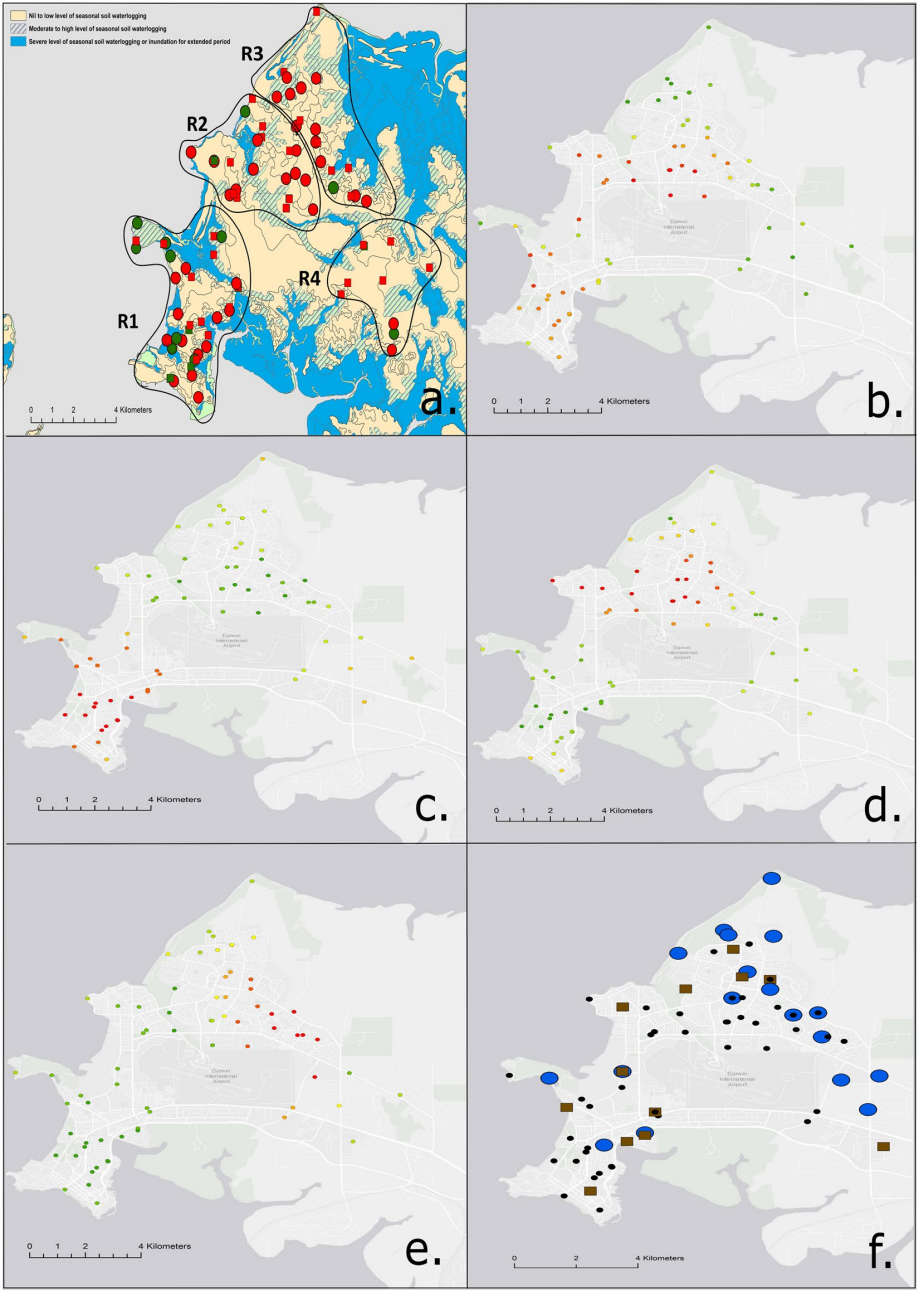
The four urban Darwin regions specified by catchment areas and direction of surface runoff at each survey site (**a**). Levels of seasonal surface runoff and areas of waterlogging are indicated by colour (yellow; nil to low levels of seasonal waterlogging, green stripes; moderate to high levels, blue; severe levels of waterlogging to fully inundated over extended period). *B. pseudomallei* positive and negative park sites (n = 45, red and green circles, respectively) and positive and negative drain sites (n = 42, red and green squares, respectively) surveyed during the 2017 rainy season (Feb-March). Getis-Ord Gi* Hot Spot Analysis (**b**–**e**). Areas with significant “hot” spots are indicated by dark red circles while “cold” spots are shown in green ((**b**) ST-36, (**c**) ST-109, (**d**) ST-327, (**e**) ST-553). Significant association of Bayes Cluster 1 with drain sites (blue circles – water and soil) as opposed to parks (brown squares - soil) (**f**). Bayes cluster 1 negative sites are denoted by black circles (ArcGIS v.10.4.1 (ESRI)).

In contrast, water was available for collection in all but six drains (36/42, 85.7%) in the second sampling round at the end of the wet season (Feb-Mar 2017). From the 108 total water samples collected 40.7% were culture and/or qPCR-positive, with 55.6% of drain sites being positive. Of these, 19.4% of samples were qPCR positive only at 38.9% sites (Table 1). From the 210 total soil samples collected from areas surrounding drains during the wet season, 30.5% were culture and/or qPCR-positive from 66.7% of sites. (Table 1, Fig. 1a).

**Table 1:** *B. pseudomallei* positive sites and samples at the end of the rainy season based on culture and qPCR detection methods.

		Drain soil	Park soil	Total soil (park and drain)	Total water (drains)
Samples	Culture positive only	5.2% (11/210)	1.5% (7/450)	2.7% (18/660)	0% (0/108)
	qPCR positive only	5.7% (12/210)	6.4% (29/450)	6.2% (41/660)	19.4% (21/108)
	Total positive by culture and/or qPCR	30.5% (64/210)	39.6% (178/450)	36.7% (242/660)	40.7% (44/108)
Sites	Total positive by culture and/or qPCR	66.7% (28/42)	82.2% (37/45)	74.7% (65/87)	55.6% (20/36)

### Incidence of *B. pseudomallei* in urban Darwin parks

From the 450 soil samples collected throughout urban parklands during the end of the rainy season, 39.6% of samples were culture and/or qPCR-positive, with 37/45 (82.2%) of surveyed sites being positive overall (Table 1, Fig. 1a).

Overall, significantly more water samples were *B. pseudomallei* positive only by qPCR compared to soil (parks and drains) (19.4% compared to 6.2%, Fisher’s exact test P < 0.001).

### Sequence type diversity

From the 135 *B. pseudomallei* environmental soil and water isolates selected for WGS we identified 35 distinct MLST genotypes (Supplementary Table S1). ST-36 was the most frequently observed molecular type (n = 42), followed by ST-109 (n = 22), ST-327 (n = 11) and ST-553 (n = 8). Eight of the ST types were novel, seven of which were identified in isolates collected from soil. Of the STs previously identified, all but one strain (ST-362) had also been isolated from a Darwin Prospective Melioidosis Study (DPMS) clinical patient^5^.

There were 24 STs that were identified in soil only, while four STs were isolated in water but not soil (ST-131, ST-456, ST-472, ST-1654). Only seven strains were isolated from both sample types, which included the four most frequently isolated STs (ST-36, ST-109, ST-144, ST-320, ST-327, ST-553, ST-616). (Supplementary Table S1). More STs were identified overall in soil than were in water (82.9% compared to 31.4% of the 35 STs recovered) (Table 2). Accounting for isolates recovered per category, drain soil had a higher overall ST diversity compared to park soil (51.2% compared to 30.1%, Fisher’s Exact test P = 0.029).

**Table 2:** Total number of samples and STs identified amongst the 135 *B. pseudomallei* study isolates based on site (42 drain and 45 park sites) and sample types.

		Drain soil	Park soil	Total soil (park and drain)	Drain water	Total drains (water and soil)
Percentage isolates (%)	Vs. total isolates examined (n = 135)	30.4% (41/135)	54.1% (73/135)	84.4% (114/135)	15.6% (21/135)	45.9% (62/135)
Percentage STs (%) identified	Vs. total STs (n = 35)	60.0% (21/35)	62.9% (22/35)	82.9% (29/35)	31.4% (11/35)	68.6% (24/35)
	Vs. number of isolates from that category	51.2% (21/41)	30.1% (22/73)	25.4% (29/114)	52.4% (11/21)	38.7% (24/62)

### Population structure of *B. pseudomallei* across urban Darwin

**Figure 2:**
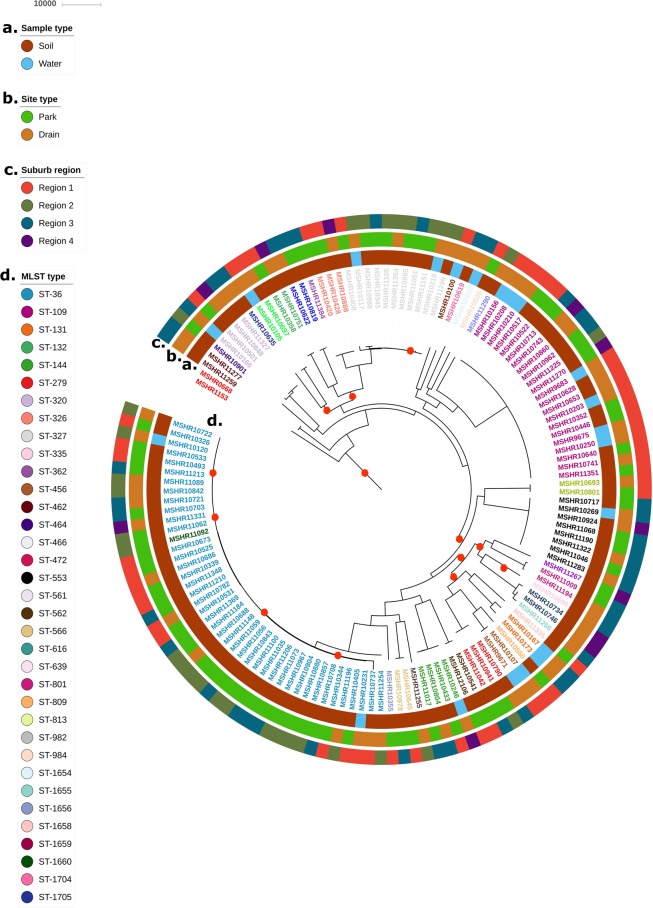
Maximum parsimony phylogeny of 137 *B. pseudomallei* genomes. MP reconstruction of 134,032 core-genome orthologous SNPs and InDels belonging to environmental survey isolates. MSHR1153 was used as the reference strain and the tree was rooted at MSHR0668. The sample type (**a**), site type (**b**), suburb region (**c**) and MLST type (**d**) of each isolate are labelled as described. Red circles on branches denote bootstrap values <80.

Despite the high levels of recombination in the *B. pseudomallei* genome, maximum parsimony (MP) phylogenetic reconstruction of the 134,032 orthologous biallelic single-nucleotide polymorphisms (SNPs) and insertion and deletion events (InDels) identified robust clustering of sequence types (Fig. 2). This was true for all but one isolate, MSHR11092, which clustered with the ST-36 group despite being designated as a novel strain type, ST-1660 (Fig. 2). However, compared to the ST-36 isolates, MSHR11092 contained a single-locus variant at the nitrite extrusion protein (narK) MLST gene loci (https://pubmlst.org/bpseudomallei/). Grouping of isolates was generally consistent with the reconstructed maximum likelihood tree^29^ (Supplementary Fig. S1).

While isolates clustered by ST, they did not group by whether they were isolated from soil or water, nor by whether they were collected from a drain or park site. There was also no observable clustering by region based on phylogenetic reconstruction of isolates.

The population structure of the 135 isolates was further defined using RhierBAPS based on a core genome SNP mapping alignment, which divided the 135 isolates into five primary sequence clusters (BAPS hierarchical level 1). These were further subdivided into 16 lineages (BAPS level 2) (Supplementary Fig S2). Three of the primary clusters at level 1, clusters 1, 2 and 3, were all comprised of multiple different STs, while ST-109 and ST-553 were uniquely assigned as cluster 4 and 5, respectively. Bayes cluster 1 contained the largest percentage of STs of any cluster (68.6%, 24/35). The five clusters were largely congruent with STs and the reconstructed phylogeny. However, clusters 1 and 2 included several STs that were distributed across different branches of the MP tree.

### *B. pseudomallei* is spatially clustered within the urban Darwin environment

There was strong evidence of spatial clustering in the environment for three of the four common urban Darwin STs for which we performed hot spot analysis, including ST-109, ST-327, and ST-553 (Getis-Ord Gi* statistic (GiZ) score >1.96, p < 0.01) (Fig. 1b–e). No significant hot spots were identified for ST-36 isolates, which were well-dispersed across sample sites (Fig. 1b). Locations and sizes of hot spots varied amongst the three STs that clustered in the environment. For ST-109, we detected a large hot spot in the city centre in the southwest region of urban Darwin (Region 1, (Fig. 1c)), while significant hot spots were observed for ST-327 (Fig. 1d) and ST-553 (Fig. 1e) in the western and eastern regions of the Darwin northern suburbs (Region 2 and Region 3, respectively). This correlated with logistic regression models of ST-109 and ST-327, which also indicated that ST-109 and ST-327 were strongly associated with Regions 1 and 2, respectively (ST-109; OR 5.7; 95% CI 1.28–25.42; p value 0.022 and ST-327; OR 7.1; 95% CI 1.53–33.30; p value 0.012). ST-553 was only isolated from the eastern northern suburb area (Region 3) (Fisher’s Exact test, p value 0.002). No hot spots were identified for any of the common STs we examined in the less densely populated Region 4 and there was strong evidence for decreased ST diversity by site in this region when compared to Regions 1 and 2 (Negative binomial regression, p value 0.024 and p value 0.006, respectively).

Hot spot analysis was also performed on the five primary RhierBAPS clusters. No significant clustering was identified for clusters 1 or 2. Likewise, since clusters 4 and 5 each contained only a single unique ST, the hot spots identified for these clusters were identical to their corresponding MLST types (ST-109- Fig. 1c, ST-553- Fig. 1e). While we observed no spatial clustering of ST-36 isolates individually (Fig. 1b) RhierBAPS cluster 3, which was comprised of ST-36, ST-566, ST-1656, ST-1659, and ST-1660, showed a significant hot spot in the central northern suburb region (Supplementary Fig. S3).

### Association of sequence types and genetic diversity with sample and site variables

*B. pseudomallei* ST-36 was strongly associated with soil (OR 5.7; 95% CI 1.82–17.8; p value 0.003) (intra-cluster correlation ICC = 0.13). This correlated with Bayes primary cluster 3 (ST-36, ST-566, ST-1656, ST-1659, and ST-1660), which was also strongly associated with soil (OR 2.6; 95% CI 1.12–5.89; p value 0.026) (ICC = 0.17). Additionally, occurrence of ST-109 was positively associated with water (OR 2.51, 95% CI; 1.0–6.32, p; 0.05). The odds to find an isolate belonging to Bayes primary cluster 1 which contained the largest ST diversity was 2.7 times larger at drain sites compared to parks (OR 2.7; 95% CI 1.23–5.94; p value 0.13) (ICC = 0.09) (Fig. 1f).

Moreover, the odds to recover more than one ST at a site was 3 times higher in parks compared to drains (OR 3.1; 95% CI 1.11–8.43; p value 0.030). Consistent with this, there was an increased probability of finding more than one ST at sites immediately adjacent to a sport’s field (OR 4.4; 95% CI 1.17–16.15; p value 0.03).

### Association of environmental ST clusters with clinical patient isolates

Lastly, the odds of having a ST-553 clinical case with patient residence in Region 3 were 4.2 times higher compared to the other Darwin regions (OR 4.2; 95% CI 2.23–7.9; p value <0.001). This matched the Region 3 cluster of ST-553 environmental survey isolates. Regional environmental clusters identified for ST-109 (Region 1) and ST-327 (Region 2) were not associated with the suspected location of infection for urban Darwin ST-109 and ST-327 clinical patient isolates.

## Discussion

In this study we analysed the distribution and local population structure of *B. pseudomallei* in urban Darwin, Northern Territory, Australia by sampling public parks and drains*. B. pseudomallei* was detected from more than 80% of park and drain sites sampled during the wet season, indicating it is ubiquitous in the urban Darwin environment and that targeting drain sites will be useful for future environmental monitoring of the bacterium. While WGS data demonstrated a large amount of genetic diversity amongst survey isolates, our results indicate that certain STs are more widespread in the urban Darwin environment. Additionally, we showed that WGS and Bayesian analysis of SNP data can be used to examine the phylogeography of *B. pseudomallei* isolates on a small geographic scale, with results suggesting there are distinct genetic populations of *B. pseudomallei* within urban Darwin and that spatial clustering of populations exists over a remarkably restricted geographical area.

While tropical soils are the known natural reservoir for *B. pseudomallei*, during periods of heavy rainfall and increased surface discharge the bacterium can be leached out of the soil and channelled into drainage areas along with other eroded particulate matter^25,26,30^. Contaminated water and movement of *B. pseudomallei* within the water table have now been established as a significant environmental source and potential distribution tool for the bacterium in melioidosis endemic regions, including southern Laos and northern Queensland, Australia^26–28^. Accordingly, our results indicate that surface runoff is a potential source of melioidosis infection within urban Darwin and may play a significant role in dispersal of *B. pseudomallei* there, both along drainage lines and via the moving water table^25,31^. Land use is known to play an integral role in the transfer of bacteria through soils to downstream aquatic systems and catchment areas^26,30,32^. As urban Darwin continues to develop and expand, increased construction may ultimately lead to additional soil erosion and runoff. This could potentially affect the distribution and dispersal of the bacterium there, particularly during periods of heavy rainfall^26,31^. Thus, the potential for increased rates of *B. pseudomallei* transmission and its propagation to uncontaminated areas should be considered as the city continues to grow. Similar considerations apply to future urban development throughout tropical northern Australia.

Previous phylogenetic investigations of *B. pseudomallei* have shown that Australian strains are ancestral to those found in Southeast Asia and globally, with the highest degree of genetic diversity identified in the Northern Territory of Australia^6,9,12,13^. Nevertheless, the nature and mechanisms for the global spread of *B. pseudomallei* remain to be elucidated, with explanations for autochthonous cases in the USA still proving elusive^33^. We identified 35 distinct *B. pseudomallei* STs as part of the investigation, further demonstrating that the bacterium is genetically diverse and well-established in the Darwin environment. Despite this, ST-36 and ST-109 comprised nearly 50% of the 135 isolates examined and ST-327 and ST-553 were the only additional molecular types for which we identified more than five isolates. These results are consistent with previous studies suggesting genetic diversity and abundance can vary amongst different *B. pseudomallei* STs and that there is an overrepresentation of a few STs within the Darwin region^6,9^. Five STs have been shown to comprise 90% of the overall clinical and environmental isolate abundance in Darwin and consistent with this, three of these were the most frequently identified molecular types in this study (ST-36, ST-109 and ST-553)^9^.

Results from this study also indicate that ST diversity can vary based on additional factors supplementary to geographic sampling area and size. We identified a significant association with increased ST diversity at park sites. Moreover, the odds of isolating more than one ST type were higher when these park sites were directly adjacent to or part of a sport’s field complex. While this finding contradicted our original hypothesis that drain catchment areas would be higher in ST diversity, previous environmental studies have shown that there is an increased occurrence of *B. pseudomallei* associated with sport’s fields^34^. This may be a consequence of optimal year-round growth conditions with higher rates of irrigation and maintained grass in the dry season, since irrigation and grass have been shown to increase the occurrence of *B. pseudomallei* in the environment^35–37^. This trend could also be associated with dissemination of the bacterium via contaminated shoes, since sport’s fields are visited by people from across the city. In line with above results, our data also showed a significant decrease in ST diversity in the outer region of the city (Region 4) compared to more urban central areas of Darwin.

Populations of *B. pseudomallei* are ecologically established and individual strain types are typically found less than 50 linear kilometres from one another in the environment^6,7,17^. Despite this, distinct genetic populations of the bacterium have only been documented across larger geographical boundaries, including the robust partitioning of Asian and Australian isolates at Wallace’s Line^7,12,13,38^. To-date, the smallest physical separation of *B. pseudomallei* populations identified has been amongst isolates from the Northern Territory, Australia and north Queensland, Australia^9^. Using high resolution WGS data and SNP-based Bayesian analysis, our results demonstrate that unique genetic populations of *B. pseudomallei* exist on an exceptionally small scale in the environment. RhierBAPS analysis of survey isolates identified five distinct populations of *B. pseudomallei*, implying there is a clear genetic distinction even within our narrow survey radius. While most clusters contained several different ST types, RhierBAPS partitioning identified two clusters each exclusively comprising only one unique ST, ST-109 or ST-553. This suggests that these two ST’s are less genetically diverse and more distantly related to the other strains we identified.

Contrary to this, Bayes Cluster 1 contained nearly 70% of the 35 STs identified, indicating some level of relatedness and shared ancestry amongst the majority of strains. Isolates that grouped within this cluster were shown to occur more frequently at drain sites. The high number of STs within this cluster could indicate an increased rate of genetic recombination at drainage catchment areas possibly related to the accumulation of *B. pseudomallei* isolates from different geographical areas, further suggesting that drains act as mechanisms of bacterial dissemination and dispersal in the environment. While these results were at odds to our previous findings indicating increased ST diversity at park sites, the latter association was observed at the site level while overall, there was a higher number of unique STs identified for drain soil isolates recovered. It is likely this trend would have been more significant had we cultured a higher percentage of qPCR-positive drain waters. However, direct PCR extraction post-enrichment has been demonstrated to be the most sensitive technique for the detection of *B. pseudomallei* in the environment compared to less-sensitive standard culture methods^39,40^. As a consequence of the high rate of qPCR-positive and low number of culture-positive drain water samples, the true ST diversity at drain sites was likely underestimated. Improved methods of culture detection and isolation of *B. pseudomallei* from water are crucial for future environmental surveys of the bacterium as determination of STs has not been possible from qPCR-positive culture-negative samples (data not shown).

Additionally, results from this study indicate that spatial clustering of *B. pseudomallei* populations can occur over a remarkably restricted geographical area, particularly for some *B. pseudomallei* genotypes. Three of the four common STs examined as part of the study were highly localised in the urban Darwin environment. While we identified no spatial clustering of ST-36, it is one of the Darwin region’s most frequently isolated molecular types and has been shown to be a significant source of melioidosis infection in the region since the Darwin Prospective Melioidosis Study (DPMS) began in 1989^5,6,9^. We also found significantly more ST-36 in soil compared to water, collectively suggesting that the ST is well-established in the urban Darwin environment and this may be why we observed no spatial clustering for the strain. Interestingly, while ST-109 is also one of the region’s most frequently identified and widely dispersed molecular types, particularly in rural Darwin which was not included in this study, we observed a significant cluster within the city urban centre^6,9,17^. Since construction and earthwork projects have substantially increased in urban central Darwin in recent years, soil disturbance may have caused the strain to resurface and spread throughout the urban area. As we identified a significant association with ST-109 and water, it is possible the ST has been spreading throughout the urban centre via drainage areas and surface water runoff following periods of heavy rainfall.

Unlike ST-36 and ST-109, ST-327 and ST-553 have both become more common in the Darwin region recently in comparison to earlier years. For example, though first isolated from a Darwin melioidosis patient in 1990, ST-553 was observed rarely over the next 20 years. However, following the heavy 2009/2010 La Niña wet season, ST-553 has become one of the region’s most frequently isolated strains both in clinical patients and the environment (unpublished data)^9^, with more limited orthologous SNP diversity observed amongst isolates in comparison to more established, widely dispersed STs^19,23^. Moreover, results from this study also demonstrate that environmental ST-553 clustering around the north-eastern suburbs (Region 3) matched the residence of clinical cases with ST-553. While it is usually not known specifically where the clinical cases acquired *B. pseudomallei*, this result suggests infection may have taken place at or near their residential address. Moreover, the observation that ST “hot spot” areas and limited ST diversity in a region are reflected by a limited ST diversity of clinical isolates in that area supports the notion of there being no succinct subset of environmental *B. pseudomallei* strains capable of causing disease^6,9^. Despite this, while the presumptive location of *B. pseudomallei* infection for clinical cases of melioidosis cases can often be speculated utilising detailed patient epidemiological history and residential address, source attribution remains subjective. Additionally, significant correlation of clinical and environmental isolates did not occur for our other three common STs. Thus, further analysis examining the geographical associations and genomic similarities between clinical and environmental isolates belonging to the same STs are needed in the future. This knowledge will allow for better understanding of melioidosis source attribution in Darwin and may help to develop public health measures mitigating against the infection in other endemic regions.

## Methods

### Environmental sites and sample collection

Forty-two drains and 45 public park areas across urban Darwin (12.5°S) were selected for the study. Apart from stratifying sites across urban Darwin, selection of drains was also based upon site accessibility. The choice of park sites ensured that 3–5 sites were surveyed across all urban Darwin suburbs. All locations chosen for the survey were managed by Darwin City Council and site approval was granted prior to the survey commencing.

Darwin is a tropical savannah environment with distinctive wet and dry seasons. During the wet season the region receives an average annual rainfall of approximately 1,700 mm (http://www.bom.gov.au/climate/data/). For drain sites, samples were collected over two sampling rounds: one round was during the 2016 build-up (Oct-Nov) to capture storm-water runoff after the first major storms of the season (total rainfall accumulated since start of the wet season <200 mm) and the second round at the end of the 2017 monsoonal wet season (Feb-March, total accumulated rainfall since start of the wet season approximately 1,100 mm) (http://www.bom.gov.au/climate/data/). Three water samples were collected from each site using one litre collection bottles attached to bottle holders where water was present (dry season; samples n = 47 (19 sites), wet season; n = 108 (36 sites)). Five soil samples were also collected in the second sampling round from areas surrounding the drain (n = 210). Where possible, soils were spaced 10 m apart and taken from 30 cm depth^41^.

For park sites, ten soil samples each were collected from 45 sites (n = 450) at the end of the 2017 monsoonal wet season (Feb-March) using a fixed interval grid sampling approach with samples spaced 10 m apart and at 30 cm depth^41^. GPS coordinates were recorded at each site using a Garmin GPS device (Garmin eTrex30).

### Environmental sample processing and confirmation

Culture of *B. pseudomallei* from soil and water was carried out using methods previously developed by Menzies School of Health Research^41–43^. Briefly, samples were enriched in Ashdown’s broth containing colistin (50 mg/L) and incubated at 37 °C aerobically for two and seven days. Enriched broth was plated onto Ashdown’s agar with gentamicin (8 mg/L) and incubated for 48 hours and colonies resembling *B. pseudomallei* were sub-cultured onto Ashdown’s agar. A maximum of ten *B. pseudomallei*-suspected colonies were sub-cultured per sample to account for genetic diversity within a sample. DNA from suspected colonies was extracted using 10% Chelex-100 resin^44^. Direct molecular detection of *B. pseudomallei* from soil and water samples was simultaneously preformed using the PowerSoil DNA isolation kit (MoBio Laboratories, USA) after an initial enrichment step of 24 h in Ashdown’s broth as previously described^39^. Confirmation of *B. pseudomallei* was done using a well-validated real-time PCR assay targeting a 115-bp segment within the type three secretion system 1 (TTS1) gene specific to the bacterium^39,45,46^.

### *B. pseudomallei* isolates included for WGS analysis

A total of 135 environmental *B. pseudomallei* strains isolated during the investigation were used in this study for WGS population analysis and geographical mapping (Supplementary Table S2). One *B. pseudomallei* soil isolate and one water isolate from each wet and dry season culture-positive drain site were initially selected for WGS (n = 40). From the parks, one *B. pseudomallei* soil isolate was selected from each culture-positive site (n = 35). All isolates were chosen at random. To examine genetic variation between sites, additional isolates cultured from all drain and park samples were screened by BOX-PCR and visualised via gel electrophoresis using methods previously described by Menzies School of Health Research^47^. One isolate was selected from every culture-positive sample within a site (min number of isolates per site, n = 1; max number of isolates examined per site, n = 9) and screened against the single site isolate already selected for WGS. All sample isolates within a site that had a different banding patterns to the primary WGS isolate were also sent for sequencing (n = 60).

### Whole-genome sequencing and sequence type (ST) assignment

Genomic DNA was extracted using the Qiagen DNeasy blood and tissue kit (Qiagen, Chadstone, Victoria, Australia) as previously described^47^. Isolates were sequenced at Australian Genome Research Facility Ltd. (Melbourne, Australia) using the Illumina HiSeq. 2500 platform (Illumina, Inc., San Diego, CA). Multi-locus sequence typing (MLST) assignment of *B. pseudomallei* environmental soil and water isolates^48^ (n = 135) was assigned from WGS data *in silico* using the Bacterial Isolates Genome Sequence database (BIGSdb) tool accessible on the *B. pseudomallei* MLST website (http://pubmlst.org/bpseudomallei/)^49^.

### Genome assembly and phylogenetic reconstruction

Orthologous core biallelic single-nucleotide polymorphisms (SNPs) and insertion and deletion events (InDels) were identified from WGS data using Genome Analysis Toolkit (GATK) in SPANDx v3.2^50^. The closed Australian *B. pseudomallei* genome MSHR1153^51^ was used as the reference for read mapping (N50:4,032,226 bp, contigs:2, total length:7,312,903 bp) and trees were rooted with MSHR0668, the most ancestral *B. pseudomallei* isolate identified to date based on a previous study^52^. A maximum-parsimony (MP) tree was constructed from 134,032 core orthologous SNPs and Indels using PAUP (v4.0a165)^53^ with 100 bootstrap replicates. A maximum-likelihood^29^ phylogenetic tree was inferred from WGS data using RAxML (v8.2.10)^54^ based on a normal model of sequence evolution using a gamma distribution. Trees were visualised in FigTree (v1.4.3) (http://tree.bio.ed.ac.uk/software/figtree/) and manipulated using Interactive Tree of Life (iTOL v4) (https://itol.embl.de)^55^.

### Hierarchical Bayesian clustering

A Bayesian approach was applied to spatially delineate and infer the genetic structure of Darwin *B. pseudomallei* isolates. Hierarchical clustering of the 135 genomes was done using RhierBAPS (version 1.1.2) implemented in R v.3.5.1 with a tree-independent approach using the core genome mapping alignment^56–58^. This method allows the population to be sub-divided into groups with closely related genetic backgrounds^59^. Clustering was performed until converging to a local optimum using two levels with k = 20 as the prior upper bound for the number of clusters. Higher levels of clustering were then performed based on the first result using k = 15 to k = 8. A value of k = 8 was chosen for both levels of clustering based on marginal likelihood values.

### Statistical analysis

Statistical analyses were computed with Stata 14.0 (www.stata.com). Generalized estimating equation (GEE) and logistic regression models with robust standard errors clustered for site (67 sites comprising 135 WGS isolates) were used to analyse whether common STs and Bayesian Clusters were associated with sample (soil or water) or site type (drain or park) and region. The four urban Darwin regions were defined by location of water catchments and which catchment area survey sites drained into. An exchangeable intra-site correlation structures (ICC) was estimated for GEE models and odds ratios (ORs) for ST or Bayesian Cluster occurrence were calculated. A negative binomial model clustered for site was used to examine whether regions, sample or site types had a higher diversity of STs or Bayesian Clusters. Categorical variables were assessed using Fisher’s Exact tests and associations with significant environmental ST clusters and clinical isolates were examined using logistic regression analysis with data grouped by region. Clinical isolate location was assigned based on individual patient epidemiology and residential address. Statistical significance was determined using a p value less than 0.05.

### Mapping of sites and spatial analysis of *B. pseudomallei* ST’s

Maps were created using ArcGIS 10.4.1 (ESRI) using GPS coordinates recorded at park and drain locations. Land unit shapefiles were obtained through the Northern Territory Government. The spatial structure of the four most frequently isolated urban Darwin STs was examined in ArcGIS implementing the Hot Spot Analysis (Getis-Ord Gi*) function with a fixed band distance. Results from the Gi* statistic were corrected for multiple testing and spatial dependence using the False Discovery Rate (FDR) correction method. Sites with GiZ scores >1.96 were considered significant at 95% confidence level (P < 0.05) and regarded as “hot” spots. GiZ scores < −2.58 indicated clustering of low values and were termed “cold” spots^60^.

### Ethical approval

This study was approved by the Human Research Ethics Committee of the NT Department of Health and the Menzies School of Health Research (HREC 02/38). Sample site approval was obtained by the Darwin City Council before commencement of the study.

## Supplementary information

Supplementary information.

## Data Availability

Raw sequence data from this study are available in the Short Read Archive in Bioproject PRJNA573745 (http://www.ncbi.nlm.nih.gov/bioproject/PRJNA573745), biosample accessions SAMN12824487-SAMN12824621).
