# Supplementary material for: Whole-genome sequencing of Burkholderia pseudomallei from an urban melioidosis hot spot reveals a fine-scale population structure and localised spatial clustering in the environment
Source: Sci Rep. 2020 Mar 25;10:5443. doi: 10.1038/s41598-020-62300-8 (PMC7096523; doi:10.1038/s41598-020-62300-8)
Supplement: Supplementary file 1 — Supplementary information. [file 41598_2020_62300_MOESM1_ESM.pdf]

Whole-genome sequencing of *Burkholderia pseudomallei* from an urban melioidosis hot-spot reveals a fine-scale population structure and localised spatial clustering in the environment

Audrey Rachlin, Mark Mayo, Jessica R. Webb, Mariana Kleinecke, Vanessa Rigas, Glenda Harrington, Bart J. Currie, and Mirjam Kaestli

Supplementary Data

Supplementary Table S1- MLST genotypes identified amongst the 135 *B. pseudomallei* isolates and numbers detected based on sample type. Asterisks denote novel STs identified as part of the survey.

ST	n (soil)	n (water)	Total
**36**	40	2	42
**109**	15	7	22
**131**	0	2	2
**132**	2	0	2
**144**	3	1	4
**279**	3	0	3
**320**	3	1	4
**326**	3	0	3
**327**	9	2	11
**335**	1	0	1
**362**	1	0	1
**456**	0	2	2
**462**	2	0	2
**464**	1	0	1
**466**	2	0	2

**472**	0	1	1
**553**	7	1	8
**561**	1	0	1
**562**	2	0	2
**566**	2	0	2
**616**	1	1	2
**639**	1	0	1
**801**	2	0	2
**809**	1	0	1
**813**	2	0	2
**982**	2	0	2
**984**	1	0	1
**1654***	0	1	1
**1655***	1	0	1
**1656***	1	0	1
**1658***	1	0	1
**1659***	1	0	1
**1660***	1	0	1
**1704***	1	0	1
**1705***	1	0	1

Supplementary Table S2- Summary of 137 *Burkholderia pseudomallei* genomes used to construct the MP and ML phylogenetic trees.

Sample ID	Species	Year	Site type	Sample source	ST	BAPS cluster	GenBank/SRA accession no.	Reference
MSHR0668	*Burkholderia pseudomallei*	1998	N/A	Human	129	1	NC_009074, NC_009075	Johnson et al., Genome Announc 2015 3(2):e00159-15
MSHR1153	*Burkholderia pseudomallei*	2001	N/A	Human	117	1	CP009271, CP009272	Johnson et al., Genome Announc 2015 3(2):e00159-15
MSHR9671	*Burkholderia pseudomallei*	2016	Drain	Water	456	1	SAMN12824487	This study
MSHR9675	*Burkholderia pseudomallei*	2016	Drain	Water	109	4	SAMN12824488	This study
MSHR9683	*Burkholderia pseudomallei*	2016	Drain	Water	109	4	SAMN12824489	This study
MSHR10100	*Burkholderia pseudomallei*	2017	Drain	Water	472	1	SAMN12824490	This study
MSHR10105	*Burkholderia pseudomallei*	2017	Drain	Water	616	1	SAMN12824491	This study
MSHR10107	*Burkholderia pseudomallei*	2017	Drain	Water	456	1	SAMN12824492	This study
MSHR10117	*Burkholderia pseudomallei*	2017	Drain	Water	327	2	SAMN12824493	This study
MSHR10120	*Burkholderia pseudomallei*	2017	Drain	Water	36	3	SAMN12824494	This study
MSHR10156	*Burkholderia pseudomallei*	2017	Drain	Water	109	4	SAMN12824495	This study
MSHR10166	*Burkholderia pseudomallei*	2017	Drain	Water	320	1	SAMN12824496	This study
MSHR10167	*Burkholderia pseudomallei*	2017	Drain	Water	131	1	SAMN12824497	This study
MSHR10173	*Burkholderia pseudomallei*	2017	Drain	Water	131	1	SAMN12824498	This study
MSHR10203	*Burkholderia pseudomallei*	2017	Drain	Water	109	4	SAMN12824499	This study
MSHR10208	*Burkholderia pseudomallei*	2017	Drain	Water	109	4	SAMN12824500	This study
MSHR10210	*Burkholderia pseudomallei*	2017	Drain	Water	109	4	SAMN12824501	This study
MSHR10218	*Burkholderia pseudomallei*	2017	Drain	Water	327	2	SAMN12824502	This study
MSHR10231	*Burkholderia pseudomallei*	2017	Drain	Water	36	3	SAMN12824503	This study
MSHR10246	*Burkholderia pseudomallei*	2017	Drain	Water	144	2	SAMN12824504	This study
MSHR10250	*Burkholderia pseudomallei*	2017	Drain	Water	109	4	SAMN12824505	This study
MSHR10259	*Burkholderia pseudomallei*	2017	Drain	Water	1654	1	SAMN12824506	This study
MSHR10269	*Burkholderia pseudomallei*	2017	Drain	Water	553	5	SAMN12824507	This study

MSHR10326	*Burkholderia pseudomallei*	2017	Park	Soil	36	3	SAMN12824508	This study
MSHR10339	*Burkholderia pseudomallei*	2017	Park	Soil	36	3	SAMN12824509	This study
MSHR10344	*Burkholderia pseudomallei*	2017	Park	Soil	36	3	SAMN12824510	This study
MSHR10352	*Burkholderia pseudomallei*	2017	Park	Soil	109	4	SAMN12824511	This study
MSHR10355	*Burkholderia pseudomallei*	2017	Park	Soil	1656	3	SAMN12824512	This study
MSHR10358	*Burkholderia pseudomallei*	2017	Park	Soil	132	1	SAMN12824513	This study
MSHR10405	*Burkholderia pseudomallei*	2017	Park	Soil	36	3	SAMN12824514	This study
MSHR10408	*Burkholderia pseudomallei*	2017	Park	Soil	327	2	SAMN12824515	This study
MSHR10420	*Burkholderia pseudomallei*	2017	Park	Soil	326	2	SAMN12824516	This study
MSHR10428	*Burkholderia pseudomallei*	2017	Park	Soil	326	2	SAMN12824517	This study
MSHR10433	*Burkholderia pseudomallei*	2017	Park	Soil	144	2	SAMN12824518	This study
MSHR10446	*Burkholderia pseudomallei*	2017	Park	Soil	109	4	SAMN12824519	This study
MSHR10493	*Burkholderia pseudomallei*	2017	Park	Soil	36	3	SAMN12824520	This study
MSHR10501	*Burkholderia pseudomallei*	2017	Park	Soil	320	1	SAMN12824521	This study
MSHR10517	*Burkholderia pseudomallei*	2017	Park	Soil	109	4	SAMN12824522	This study
MSHR10522	*Burkholderia pseudomallei*	2017	Park	Soil	109	4	SAMN12824523	This study
MSHR10525	*Burkholderia pseudomallei*	2017	Park	Soil	36	3	SAMN12824524	This study
MSHR10526	*Burkholderia pseudomallei*	2017	Park	Soil	984	1	SAMN12824525	This study
MSHR10531	*Burkholderia pseudomallei*	2017	Park	Soil	36	3	SAMN12824526	This study
MSHR10533	*Burkholderia pseudomallei*	2017	Park	Soil	36	3	SAMN12824527	This study
MSHR10541	*Burkholderia pseudomallei*	2017	Park	Soil	562	1	SAMN12824528	This study
MSHR10550	*Burkholderia pseudomallei*	2017	Park	Soil	809	1	SAMN12824529	This study
MSHR10619	*Burkholderia pseudomallei*	2017	Drain	Soil	1704	1	SAMN12824530	This study
MSHR10622	*Burkholderia pseudomallei*	2017	Drain	Soil	466	1	SAMN12824531	This study
MSHR10628	*Burkholderia pseudomallei*	2017	Drain	Soil	109	4	SAMN12824532	This study
MSHR10635	*Burkholderia pseudomallei*	2017	Drain	Soil	1705	1	SAMN12824533	This study
MSHR10640	*Burkholderia pseudomallei*	2017	Park	Soil	109	4	SAMN12824534	This study
MSHR10645	*Burkholderia pseudomallei*	2017	Park	Soil	566	3	SAMN12824535	This study

MSHR10653	*Burkholderia pseudomallei*	2017	Drain	Soil	109	4	SAMN12824536	This study
MSHR10673	*Burkholderia pseudomallei*	2017	Park	Soil	36	3	SAMN12824537	This study
MSHR10686	*Burkholderia pseudomallei*	2017	Drain	Soil	36	3	SAMN12824538	This study
MSHR10688	*Burkholderia pseudomallei*	2017	Drain	Soil	36	3	SAMN12824539	This study
MSHR10690	*Burkholderia pseudomallei*	2017	Drain	Soil	335	1	SAMN12824540	This study
MSHR10693	*Burkholderia pseudomallei*	2017	Drain	Soil	813	1	SAMN12824541	This study
MSHR10697	*Burkholderia pseudomallei*	2017	Drain	Soil	616	1	SAMN12824542	This study
MSHR10703	*Burkholderia pseudomallei*	2017	Park	Soil	36	3	SAMN12824543	This study
MSHR10708	*Burkholderia pseudomallei*	2017	Park	Soil	36	3	SAMN12824544	This study
MSHR10713	*Burkholderia pseudomallei*	2017	Park	Soil	109	4	SAMN12824545	This study
MSHR10717	*Burkholderia pseudomallei*	2017	Park	Soil	553	5	SAMN12824546	This study
MSHR10721	*Burkholderia pseudomallei*	2017	Drain	Soil	36	3	SAMN12824547	This study
MSHR10722	*Burkholderia pseudomallei*	2017	Drain	Soil	36	3	SAMN12824548	This study
MSHR10734	*Burkholderia pseudomallei*	2017	Drain	Soil	982	1	SAMN12824549	This study
MSHR10737	*Burkholderia pseudomallei*	2017	Drain	Soil	36	3	SAMN12824550	This study
MSHR10741	*Burkholderia pseudomallei*	2017	Drain	Soil	109	4	SAMN12824551	This study
MSHR10743	*Burkholderia pseudomallei*	2017	Drain	Soil	109	4	SAMN12824552	This study
MSHR10746	*Burkholderia pseudomallei*	2017	Drain	Soil	982	1	SAMN12824553	This study
MSHR10751	*Burkholderia pseudomallei*	2017	Drain	Soil	132	1	SAMN12824554	This study
MSHR10782	*Burkholderia pseudomallei*	2017	Drain	Soil	36	3	SAMN12824555	This study
MSHR10790	*Burkholderia pseudomallei*	2017	Drain	Soil	279	1	SAMN12824556	This study
MSHR10801	*Burkholderia pseudomallei*	2017	Park	Soil	813	1	SAMN12824557	This study
MSHR10804	*Burkholderia pseudomallei*	2017	Drain	Soil	144	2	SAMN12824558	This study
MSHR10819	*Burkholderia pseudomallei*	2017	Park	Soil	466	1	SAMN12824559	This study
MSHR10842	*Burkholderia pseudomallei*	2017	Drain	Soil	36	3	SAMN12824560	This study
MSHR10843	*Burkholderia pseudomallei*	2017	Park	Soil	36	3	SAMN12824561	This study
MSHR10848	*Burkholderia pseudomallei*	2017	Drain	Soil	320	1	SAMN12824562	This study
MSHR10857	*Burkholderia pseudomallei*	2017	Park	Soil	36	3	SAMN12824563	This study

MSHR10860	*Burkholderia pseudomallei*	2017	Drain	Soil	109	4	SAMN12824564	This study
MSHR10880	*Burkholderia pseudomallei*	2017	Park	Soil	36	3	SAMN12824565	This study
MSHR10888	*Burkholderia pseudomallei*	2017	Drain	Soil	326	2	SAMN12824566	This study
MSHR10901	*Burkholderia pseudomallei*	2017	Park	Soil	464	1	SAMN12824567	This study
MSHR10904	*Burkholderia pseudomallei*	2017	Park	Soil	36	3	SAMN12824568	This study
MSHR10924	*Burkholderia pseudomallei*	2017	Drain	Soil	553	5	SAMN12824569	This study
MSHR10941	*Burkholderia pseudomallei*	2017	Park	Soil	279	1	SAMN12824570	This study
MSHR10945	*Burkholderia pseudomallei*	2017	Park	Soil	327	2	SAMN12824571	This study
MSHR10962	*Burkholderia pseudomallei*	2017	Park	Soil	109	4	SAMN12824572	This study
MSHR10967	*Burkholderia pseudomallei*	2017	Park	Soil	36	3	SAMN12824573	This study
MSHR10978	*Burkholderia pseudomallei*	2017	Park	Soil	566	3	SAMN12824574	This study
MSHR10988	*Burkholderia pseudomallei*	2017	Park	Soil	327	2	SAMN12824575	This study
MSHR10995	*Burkholderia pseudomallei*	2017	Park	Soil	327	2	SAMN12824576	This study
MSHR11009	*Burkholderia pseudomallei*	2017	Park	Soil	801	1	SAMN12824577	This study
MSHR11017	*Burkholderia pseudomallei*	2017	Park	Soil	144	2	SAMN12824578	This study
MSHR11035	*Burkholderia pseudomallei*	2017	Park	Soil	36	3	SAMN12824579	This study
MSHR11042	*Burkholderia pseudomallei*	2017	Park	Soil	279	1	SAMN12824580	This study
MSHR11046	*Burkholderia pseudomallei*	2017	Park	Soil	553	5	SAMN12824581	This study
MSHR11051	*Burkholderia pseudomallei*	2017	Park	Soil	327	2	SAMN12824582	This study
MSHR11056	*Burkholderia pseudomallei*	2017	Park	Soil	36	3	SAMN12824583	This study
MSHR11059	*Burkholderia pseudomallei*	2017	Park	Soil	36	3	SAMN12824584	This study
MSHR11062	*Burkholderia pseudomallei*	2017	Park	Soil	36	3	SAMN12824585	This study
MSHR11068	*Burkholderia pseudomallei*	2017	Park	Soil	553	5	SAMN12824586	This study
MSHR11073	*Burkholderia pseudomallei*	2017	Park	Soil	36	3	SAMN12824587	This study
MSHR11089	*Burkholderia pseudomallei*	2017	Drain	Soil	36	3	SAMN12824588	This study
MSHR11092	*Burkholderia pseudomallei*	2017	Park	Soil	1660	3	SAMN12824589	This study
MSHR11100	*Burkholderia pseudomallei*	2017	Park	Soil	36	3	SAMN12824590	This study
MSHR11105	*Burkholderia pseudomallei*	2017	Park	Soil	327	2	SAMN12824591	This study

MSHR11148	*Burkholderia pseudomallei*	2017	Park	Soil	36	3	SAMN12824592	This study
MSHR11151	*Burkholderia pseudomallei*	2017	Park	Soil	327	2	SAMN12824593	This study
MSHR11184	*Burkholderia pseudomallei*	2017	Park	Soil	36	3	SAMN12824594	This study
MSHR11190	*Burkholderia pseudomallei*	2017	Park	Soil	553	5	SAMN12824595	This study
MSHR11194	*Burkholderia pseudomallei*	2017	Drain	Soil	801	1	SAMN12824596	This study
MSHR11196	*Burkholderia pseudomallei*	2017	Drain	Soil	36	3	SAMN12824597	This study
MSHR11199	*Burkholderia pseudomallei*	2017	Drain	Soil	327	2	SAMN12824598	This study
MSHR11206	*Burkholderia pseudomallei*	2017	Park	Soil	36	3	SAMN12824599	This study
MSHR11210	*Burkholderia pseudomallei*	2017	Park	Soil	36	3	SAMN12824600	This study
MSHR11213	*Burkholderia pseudomallei*	2017	Park	Soil	36	3	SAMN12824601	This study
MSHR11225	*Burkholderia pseudomallei*	2017	Park	Soil	109	4	SAMN12824602	This study
MSHR11254	*Burkholderia pseudomallei*	2017	Drain	Soil	36	3	SAMN12824603	This study
MSHR11255	*Burkholderia pseudomallei*	2017	Drain	Soil	1659	3	SAMN12824604	This study
MSHR11259	*Burkholderia pseudomallei*	2017	Drain	Soil	462	1	SAMN12824605	This study
MSHR11266	*Burkholderia pseudomallei*	2017	Drain	Soil	1655	1	SAMN12824606	This study
MSHR11267	*Burkholderia pseudomallei*	2017	Park	Soil	639	1	SAMN12824607	This study
MSHR11270	*Burkholderia pseudomallei*	2017	Park	Soil	109	4	SAMN12824608	This study
MSHR11277	*Burkholderia pseudomallei*	2017	Drain	Soil	462	1	SAMN12824609	This study
MSHR11283	*Burkholderia pseudomallei*	2017	Drain	Soil	553	5	SAMN12824610	This study
MSHR11290	*Burkholderia pseudomallei*	2017	Drain	Soil	561	1	SAMN12824611	This study
MSHR11322	*Burkholderia pseudomallei*	2017	Drain	Soil	553	5	SAMN12824612	This study
MSHR11327	*Burkholderia pseudomallei*	2017	Drain	Soil	320	1	SAMN12824613	This study
MSHR11331	*Burkholderia pseudomallei*	2017	Drain	Soil	36	3	SAMN12824614	This study
MSHR11335	*Burkholderia pseudomallei*	2017	Park	Soil	1658	1	SAMN12824615	This study
MSHR11348	*Burkholderia pseudomallei*	2017	Park	Soil	36	3	SAMN12824616	This study
MSHR11351	*Burkholderia pseudomallei*	2017	Park	Soil	109	4	SAMN12824617	This study
MSHR11354	*Burkholderia pseudomallei*	2017	Drain	Soil	327	2	SAMN12824618	This study
MSHR11369	*Burkholderia pseudomallei*	2017	Park	Soil	36	3	SAMN12824619	This study

MSHR11384	*Burkholderia pseudomallei*	2017	Park	Soil	362	2	SAMN12824620	This study
MSHR12106	*Burkholderia pseudomallei*	2017	Park	Soil	562	1	SAMN12824621	This study

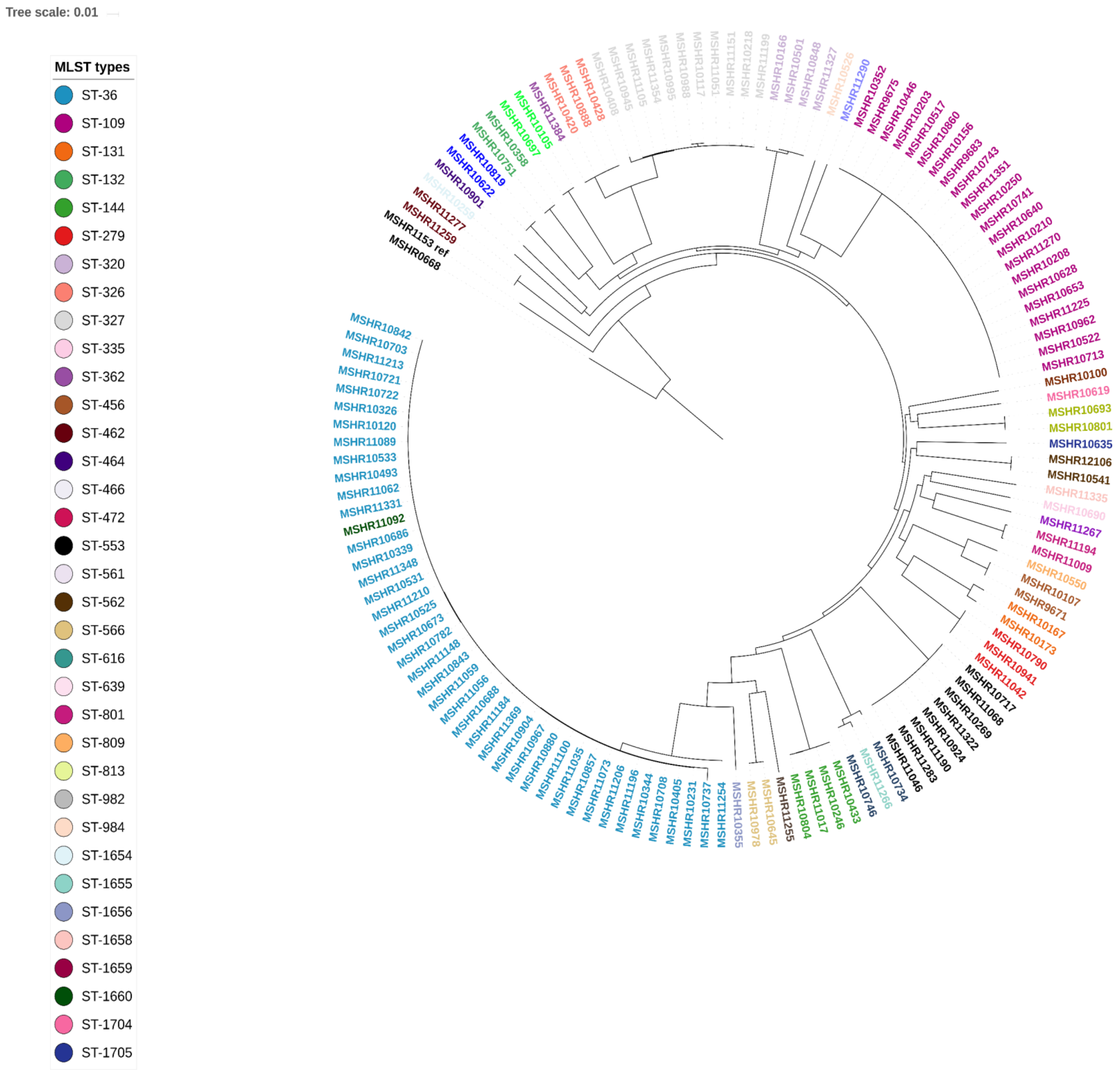

Supplementary Figure S1

Maximum likelihood phylogeny of 137 *B. pseudomallei* genomes derived using RAxML. MLST strain types are coded by colour.

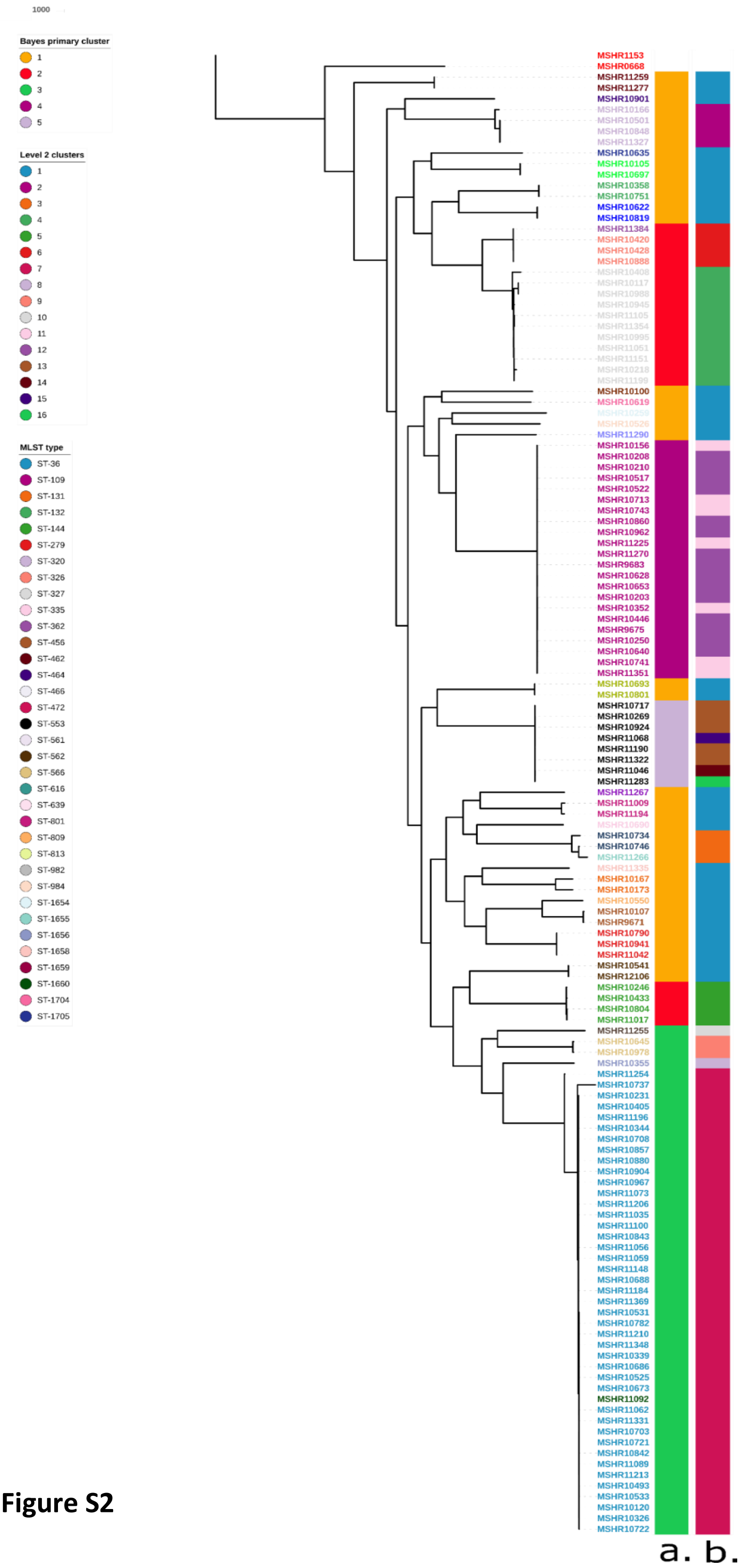

Supplementary Figure S2

Maximum parsimony phylogeny of 137 *B. pseudomallei* genomes constructed using 134,032 core-genome orthologous SNPs and InDels. BAPS level 1 primary clusters (a.) and level 2 clusters (b.) were inferred using RhierBAPS and are colour-coded next to respective strains.

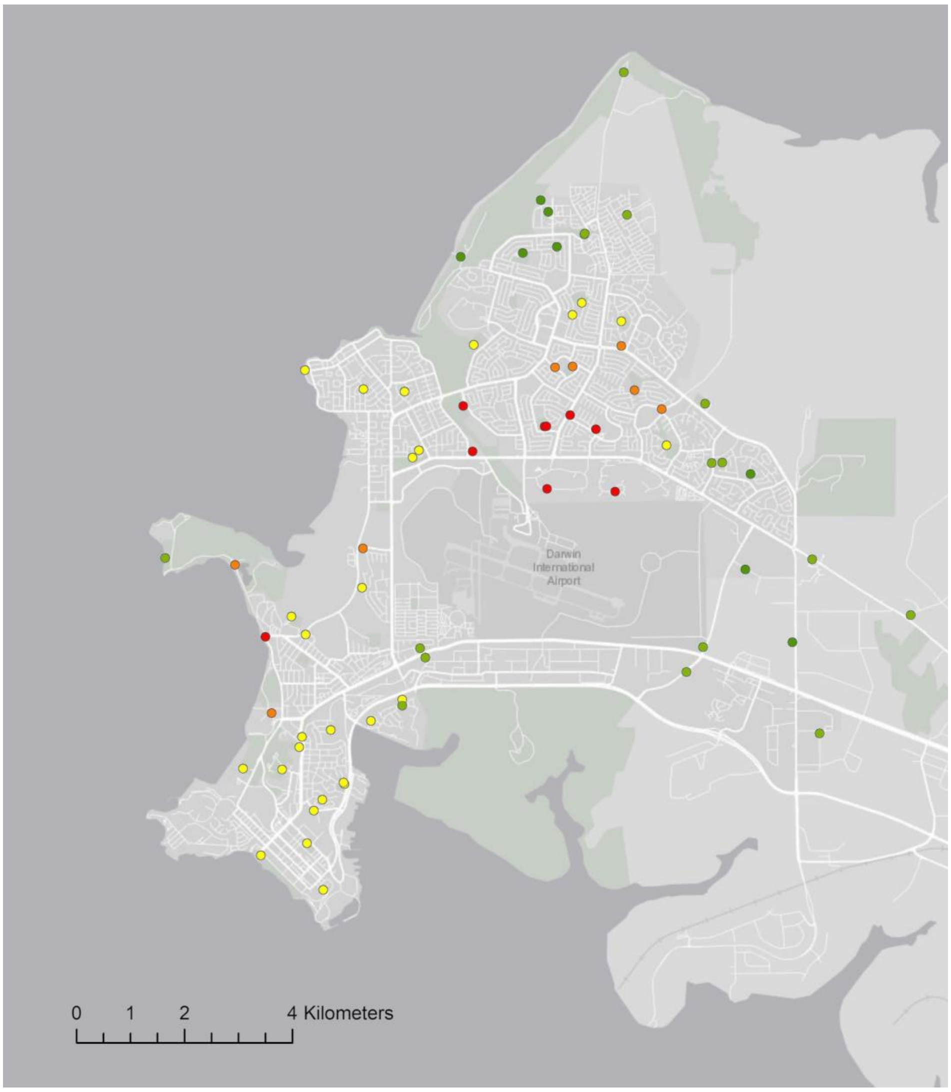

Supplementary Figure S3- Getis-Ord Gi* Hot Spot Analysis of Bayes cluster 3. Areas with significant “hot” spots are indicated by dark red circles while “cold” spots are shown in green (ArcGIS v.10.4.1 (ESRI)).
